# Management of depression during the perinatal period: state of the evidence

**DOI:** 10.1186/s13033-022-00531-0

**Published:** 2022-04-25

**Authors:** Idan Falek, Mary Acri, Joanna Dominguez, Jennifer Havens, Mary McCord, Sarah Sisco, Wendy Wilcox, Kimberly Hoagwood

**Affiliations:** 1 Department of Child & Adolescent Psychiatry, NYU School of Medicine, One Park Avenue, 7th Floor, New York, NY 10016 USA; 2 New York City Health and Hospitals Corporation, 55 Water St., New York, NY 10041 USA

**Keywords:** Perinatal depression, Guidelines for management, Pathway to care, Obstetric care, Pediatric care

## Abstract

**Background:**

Perinatal depression (PND) is a prevalent ailment that affects both the woman and her family. Addressing PND in primary health care, such as pediatrics and obstetric care settings, has been proposed as an effective way to identify and treat women.

**Objective:**

The purpose of this study is to examine best practices for management of PND in obstetric and pediatric settings, as well as investigate the evidence that supports the guidelines.

**Methods:**

Guidelines were identified through a literature search and discussion with experts in the field of perinatal depression, while evidence was examined through a literature search of reviews and thereafter experimental studies.

**Results:**

Twenty-five guidelines, across 17 organizations were retained for analysis. Findings suggest that there is little or varied guidance on the management of PND, as well as a lack of specificity. Treatment was the topic most frequently reported, followed by screening. However best practices vary greatly and often contradict one another. Across all areas, there is inadequate or contrasting evidence to support these guidelines.

**Conclusions:**

Although there was consensus on the key steps in the pathway to care, the review revealed lack of consensus across guidelines on specific issues relating to identification and management of depression during the perinatal period. Clinicians may use these recommendations to guide their practice, but they should be aware of the limitations of the evidence supporting these guidelines and remain alert to new evidence. There is a clear need for researchers and policymakers to prioritize this area in order to develop evidence-based guidelines for managing perinatal depression.

## Introduction

Approximately one in every ten women experiences major depression within their lifetime [4, 77], which is twice as high as rates of depression amongst men [5, 17, 39]. The perinatal period, which encompasses pregnancy and the postpartum period, is one of the most vulnerable time points for the onset of depression among women [15]. In one systematic review across varying countries, 7.4% of women met criteria for depression during the first trimester, 12.8% met criteria during the second trimester, and 12.0% during the third [13], while between 10 and 15% of women have been found to experience depression during the postpartum period [9, 35].

The entire family is impacted by perinatal depression (PND). In addition to being one of the leading causes of disability amongst adults and an accelerant to poor health and premature mortality [18, 31, 75], infants born to women experiencing depression have a heightened risk for low birth weight and premature delivery, and demonstrate worse cognitive performance, behavior problems, and heightened anger [14, 71]. Caregiver depression also undermines the quality of parenting, resulting in decreased parental involvement and warmth and an increased risk of child maltreatment [25, 45].

Identifying PND within health settings has been proposed as an effective way to facilitate detection and treatment [40, 51]. The American Academy of Pediatrics [8], for example, advises screening mothers in pediatric settings at 1, 2, 4, and 6 months post-delivery, which correspond to their child’s well-child visit [29]. Integrating mental health services into health settings reduces logistical barriers to access [61], provides frequent and consistent contact [40, 51], and potentially decreases perceptual barriers such as stigma [61, 69]. To this end, several recent studies have found that female caregivers preferred and were more likely to seek mental health treatment when offered in primary care and obstetric settings [32, 51].

There are significant barriers to addressing PND in health settings, however; providers cite concerns about being too busy to screen, not feeling confident addressing maternal depression, and lacking knowledge about available mental health resources to refer caregivers to [21, 28, 30, 34, 72]. To this end, Lancaster et al. [40] found less than half of obstetricians reported that residency prepared them to diagnose depression. Consequently, most women with perinatal depression are not identified, in one study, only 26% of pregnant women who met criteria for depression or anxiety were identified by their obstetrics provider as having a mental health problem [66]. Further, most women do not receive mental health services at all for any mental health condition [1, 24, 62], with a systematic review finding that 8.6% of women with antenatal depression and 6.3% of women with postpartum depression received adequate treatment [24]. This is further evidenced by Kessler et al. [37] national epidemiology study of major depressive disorder (MDD) finding that 51.6% of 12-month cases received health care treatment for MDD, with 21.7% of 12-month cases being adequate treated. A recent analysis of the National Survey on Drug Use and Health, for example, found pregnant women were statistically less likely to receive treatment for depression than women who were not pregnant, and their perceived unmet need was statistically greater irrespective of whether they received treatment [62]. Coalescing best practices for the management of PND in health settings can benefit providers who may not feel equipped or lack knowledge about how to identify and address of depression during a particularly vulnerable period in the health and wellbeing of families. It may also be valuable to compare guidelines from different sources to examine where they may overlap and diverge to improve management practice. Accordingly, this paper aims to review and synthesize best practice guidelines in addressing perinatal depression in health settings. We review and analyze guidelines from national and international sources, covering 1990 to 2021, compare them to current practice, and make recommendations for strengthening both the evidence base and its implementation to improve maternal and familial health.

## Methods

To identify guidelines and evidence from the empirical literature, two separate steps were undertaken: (1) a search of guidelines, and (2) a review of the literature to examine the evidence. In order to conduct a comprehensive search of guidelines, three methods were employed: (1) a search of the Guideline International Network using the term depression, (2) website and Google searches, and (3) discussions with experts in the field of perinatal depression.

## Inclusion and exclusion criteria

Guidelines were included if they:

1. Described the management of perinatal depression (corresponding to the period of pregnancy and/or the first 12 months after childbirth, although the postpartum period may vary across sources) in pediatrics or obstetric care settings,
2. Were published between 1990 and 2021, and
3. Were written in English.

**Fig. 1:**
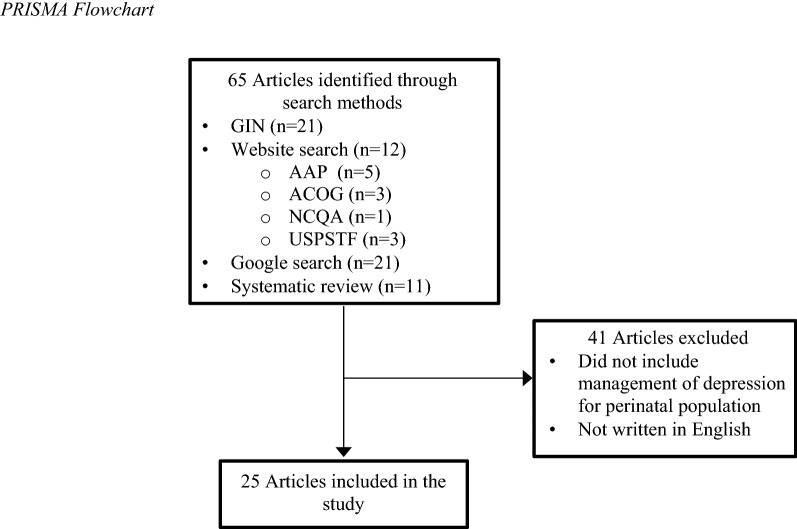
PRISMA flowchart. *AAP* American Academy of Pediatrics; ACOG *American* College of Obstetricians and Gynecologists; *GIN* Guideline International Network; *NCQA* National Committee for Quality Assurance; *USPSTF* United States Preventive Services Task Force

A total of 65 guidelines were retrieved; of these, 25 met the above criteria and were included in this study. See Figure 1.

Next, we reviewed the research literature to examine evidence for the management of perinatal depression as described by the guidelines. To locate this literature, we undertook an online search using Google Scholar and PubMed databases with search limits restricted by year (1990–2021) and language (English). Search terms used for the literature review included two categories: perinatal period (pregnancy, postpartum) and depression management (screening, assessment to confirm the diagnosis, suicide assessment, treatment, reassessment, remission, treatment of refractory depression and care management). Search terms within the perinatal period category were linked with “or”, and the perinatal period and one of the depression management categories were linked with “and” to capture studies that included at least one search term from both the first and second categories. If meta-analyses and systematic reviews were identified, the search was ended. If none were found, the search was expanded to include individual experimental, quasi-experimental, and non-experimental studies.

## Results

The 25 guidelines were published across 17 organizations, with six organizations publishing multiple guidelines. More than half of the guidelines (n = 14, 56%) were issued by international bodies; the remaining 11 (44%) were issued by the United States. The international guidelines included in the reviews spanned multiple regions as defined by the World Bank [74], including Europe and Central Asia (n = 6, 43%; United Kingdom, Netherlands, Spain, and Nordic region [Denmark, Finland, Iceland, Norway, Sweden]), East Asia and Pacific (n = 5, 36%; Australia, New Zealand, and Singapore), and North America (n = 3, 21%; Canada). National guidelines were published by a variety of organizations including American Academy of Pediatrics (AAP), American College of Obstetricians and Gynecologists (ACOG), United States Preventive Services Task Force (USPSTF), Postpartum Support International (PSI), National Committee for Quality Assurance (NCQA), and Kaiser Permanente. Guidelines were published between 2009 and 2020, with more than a quarter (n = 7, 28%) being published in 2018. See Table 1.

**Table 1:** Perinatal depression guidelines

Author/year	Organization	Region (country)
1. Alhusen and Alvarez [6]	Wolters Kluwer Health (WKH)	Europe and Central Asia (Netherlands)
2. Álvarez Ariza et al. [7]	Spain Ministry of Health, Social Services and Equality	Europe and Central Asia (Spain)
3. Austin et al. [11]	National Health and Medical Research Council (NHMRC) and Beyond Blue	East Asia and Pacific (Australia)
4. Austin et al. [10]	National Health and Medical Research Council (NHMRC) and Centre of Perinatal Excellence (COPE)	East Asia and Pacific (Australia)
5. Brock [16]	National Committee for Quality Assurance (NCQA)	North America (United States)
6. Chua et al. [22]	Singapore Ministry of Health	East Asia and Pacific (Singapore)
7. Committee on Obstetric Practice [23]	American College of Obstetricians and Gynecologists (ACOG)	North America (United States)
8. Curry et al. [26]	United States Preventive Services Task Force (USPSTF)	North America (United States)
9. Diaz and Plunkett [27]	American Academy of Pediatrics (AAP)	North America (United States)
10. Earls et al. [29]	American Academy of Pediatrics (AAP)	North America (United States)
11. LeFevre et al. [41]	United States Preventive Services Task Force (USPSTF)	North America (United States)
12. MacQueen et al. [46]	Canadian Network for Mood and Anxiety Treatments (CANMAT)	North America (Canada)
13. Molenaar et al. [50]	Royal Australian and New Zealand College of Obstetricians and Gynaecologists (RANZCOG)	
14. National Institute for Health and Care Excellence [52]	National Institute for Health and Care Excellence (NICE)	Europe and Central Asia (United Kingdom)
15. Nordeng and Jettestad [53]	Nordic Federation of Obstetrics and Gynecology (NFOG)	Europe and Central Asia [Nordic region (Denmark, Finland, Iceland, Norway, Sweden)]
16. O’Hara and Engeldinger [55]	Wolters Kluwer Health (WKH)	Europe and Central Asia (Netherlands)
17. Postpartum Support International [56]	Postpartum Support International (PSI)	North America (United States)
18. Rafferty et al. [57]	American Academy of Pediatrics (AAP)	North America (United States)
19. Registered Nurses’ Association of Ontario [58]	Registered Nurses’ Association of Ontario (RNAO)	North America (Canada)
20. Robson et al. [59]	Royal Australian and New Zealand College of Obstetricians and Gynaecologists (RANZCOG)	East Asia and Pacific (Australia and New Zealand)
21. Scottish Intercollegiate Guidelines Network [63]	Scottish Intercollegiate Guidelines Network (SIGN)	Europe and Central Asia (United Kingdom)
22. Siu et al. [65]	United States Preventive Services Task Force (USPSTF)	North America (United States)
23. Sparks et al. [68]	Kaiser Permanente	North America (United States)
24. Williams et al. [73]	British Columbia Ministry of Health	North America (Canada)
25. Yonkers et al. [76]	American College of Obstetricians and Gynecologists (ACOG)	North America (United States)

Following previous guideline reviews of depression [44], results were explored by the following categories: screening, assessment to confirm the diagnosis, suicide assessment, treatment, reassessment, remission, treatment of refractory depression and care management. See Table 2.

**Table 2:** Guideline recommendations

Guideline	Screening	Assessment to confirm diagnosis	Suicide assessment	Treatment	Reassessment	Remission	Treatment refractory depression	Care management
1. Alhusen and Alvarez [6]	X			X				X
2. Álvarez Ariza et al. [7]			X				X	X
3. Austin et al. [11]	X	X	X	X			X	X
4. Austin et al. [10]	X	X	X	X	X		X	X
5. Brock [16]				X				
6. Chua et al. [22]	X			X			X	
7. Committee on Obstetric Practice [23]	X		X	X				X
8. Curry et al. [26]	X			X				X
9. Diaz and Plunkett [27]	X	X						
10. Earls et al. [29]	X	X	X	X				X
11. LeFevre al. [41]			X					
12. MacQueen et al. [46]				X			X	
13. Molenaar et al. [50]				X				
14. NICE [52]	X	X	X	X				
15. Nordeng and Jettestad [53]	X			X			X	
16. O’Hara and Engeldinger [55]	X			X	X		X	X
17. PSI [56]	X							X
18. Rafferty et al. [57]	X	X	X	X			X	X
19. RNAO [58]	X	X	X	X			X	
20. Robson et al. [59]		X		X	X			X
21. SIGN [63]	X		X	X				
22. Siu et al. [65]	X			X		X	X	
23. Sparks et al. [68]	X		X	X		X		X
24. Williams et al. [73]	X	X	X	X			X	
25. Yonkers et al. [76]			X	X			X	X

## Screening

### Guideline Recommendations

The majority of guidelines (n = 18, 72%) issued recommendations on screening for PND, and of them 16 (89%) provided multiple recommendations.

#### Screening timeframe

Fourteen guidelines (78%) recommended routine screening during the perinatal period. Of them, eight (57%) recommended screening during both the prenatal and postpartum period. Half (n = 4, 50%) of the guidelines provided specific screening intervals that permitted the integration of screening with existing routine care, allowing the mother who initially may not be comfortable to disclose to do so at a later date, maximizing  the opportunity to engage if they miss a visit, and reducing false-positive and false-negative results [27, 29, 57]. Two guidelines (11%) stated that because the evidence was inconsistent, an optimal screening interval was unknown and that no recommendations regarding specific frequency and timing could be made.

#### Setting

More than half of the guidelines (n = 12, 67%) recommended screening locations; of them, seven (58%) recommended screening in obstetric settings, three (25%) recommended screening in pediatric settings, and two (17%) recommended screening in both obstetric and pediatric settings. These settings were identified as optimal because screenings could be readily integrated with existing care, and permitted fostering a longitudinal relationship between providers and women, which facilitated building trust and may in turn lead to disclosure of depressive symptoms [11, 27, 29, 55].

#### Instrumentation

Twelve guidelines (67%) also recommended specific screening instruments, with six guidelines (50%) recommending multiple instruments including the Edinburgh Postnatal Depression Scale (EPDS; n = 8, 67%), and the two and nine-question versions of the Patient Health Questionnaire (PHQ-9; n = 3, 12%; PHQ-2; n = 3, 12%). One guideline (6%) cited that there were no accurate screening tools for identifying who is at risk of perinatal depression, as more data were needed before incorporating perinatal risk factors into the screening tools. Variability was also found in clinical cutoffs for each instrument, although not all guidelines provided cutoff scores. See Table 3.

**Table 3:** Screening guidelines

Guideline	Perinatal period	Time interval	Setting	Instrumentation (clinical cutoff score)
1. Alhusen and Alvarez [6]	•Postpartum period	•1-, 2- and 4-month WCV	•Pediatrics	N/A
2. Austin et al. [11]	•Pregnancy and postpartum period	•Twice in pregnancy (earliest possible in pregnancy and repeat) •Twice postpartum (6–12 weeks after birth and repeat once in first postnatal year)	•Obstetrics	•EPDS (10–12) •Psychosocial assessment
3. Austin et al. [10]	•Pregnancy and postpartum period	•At least once, preferably twice, in pregnancy •At least once, preferably twice, postnatal period (ideally 6–12 weeks after the birth)	•Obstetrics	•EPDS (10–12)
4. Chua et al. [22]	N/A	N/A	N/A	•PHQ-2 (unreported)
5. Committee on Obstetric Practice [23]	•Postpartum period	N/A	•Obstetrics	•Psychosocial assessment
6. Curry et al. [26]	•Postpartum period	N/A	N/A	•No accurate screening tools
7. Diaz and Plunkett [27]	•Pregnancy and postpartum period	•1-, 2-, 4- and 6-month WCV	•Obstetrics and pediatric	•EPDS (9.5–12.5) •PHQ-9 (unreported) •PHQ-2 (unreported)
8. Earls et al. [29]	•Postpartum period	•1-, 2-, 4- and 6-month WCV	•Pediatrics	N/A
9. NICE [52]	•Pregnancy and postpartum period	N/A	•Obstetrics	•PHQ-2 (unreported)
10. Nordeng and Jettestad [53]	N/A	N/A	N/A	•EPDS (unreported) •MADRS (unreported)
11. O’Hara and Engeldinger [55]	•Pregnancy and postpartum period	•At least once in pregnancy •After delivery or at the 6-week follow-up appointment	•Obstetrics	N/A
12. PSI [56]	•Pregnancy and postpartum period	N/A	N/A	•EPDS (10) •PHQ-9 (10)
13. Rafferty et al. [57]	•Postpartum period	N/A	•Pediatrics	N/A
14. RNAO [58]	N/A	•Optimal screening intervals are unknown	N/A	N/A
15. SIGN [63]	•Pregnancy and postpartum period	N/A	•Obstetrics	•EPDS (9/10 or 12/13) •Whooley Questions (unreported)
16. Siu et al. [65]	N/A	•Optimal screening intervals are unknown	N/A	•EPDS (unreported) •PHQ-9 (unreported) •GDS (unreported) •HADS (unreported)
17. Sparks et al. [68]	•Pregnancy and postpartum period	•Four times in pregnancy (first prenatal visit, 16-weeks, 32-weeks, postpartum) •Four times in postpartum (7–14-day WCV, 4-, 6- and 12-month WCV)	•Obstetrics and pediatric	•MBH (unreported)
18. Williams et al. [73]	•Pregnancy	•28- and 32-weeks' gestation	•Obstetrics	•EPDS (9–14)

*EPDS* Edinburgh Postnatal Depression Scale; *GDS* Geriatric Depression Scale; *HADS* Hospital Anxiety and Depression Scale; *MBH* Maternal Behavioral Health Screening; *MADRS* Montgomery-Asberg Depression Rating; *PHQ* Patient Health Questionnaire; *WCV* well child visit

### Summary of literature review

A recent review for the United States Preventive Services Task Force (USPSTF), which synthesized 33 studies, found that screening women during the perinatal period reduced the prevalence of depression [54]. Notably, the review cited limitations of a relatively small number of studies, few trials with good applicability to primary care, and many studies with very small sample sizes. These limitations were further recognized in Milgrom and Gemmill’s [47] review of an undisclosed number of studies, in which they found that high-quality evidence about the clinical effectiveness of screening programs was slow to emerge, with relevant studies being rare and having small samples. No trials tested the effectiveness of screening in reducing perinatal morbidity were of high enough quality to meaningfully inform clinical practice [47]. Additionally, there was an insufficient quality and quantity of available evidence to establish the optimal time intervals to screen [47]. The lack of sufficient evidence was reinforced in Ukatu et al.’s [70] review of 68 articles on postpartum depression (PPD) screening tools. Findings suggested that no instrument could be deemed best at accurately detecting PPD on the basis of sensitivity and specificity, and there was no evidence for a recommended time duration in which screening should be completed.

## Assessment to confirm diagnosis

### Guideline recommendations

Slightly over one-third of the guidelines (n = 9, 36%) recommended conducting an assessment to confirm depression after screening. Three guidelines (33%) made multiple recommendations. The remaining guidelines (n = 16, 64%) did not reference confirmation of a diagnosis post-screening.

#### Assessment by severity

Six of the nine guidelines (67%) made recommendations to confirm a diagnosis based on severity. In less severe situations, which were defined as depression without suicidal ideation or risk of harm, a score of 10–12 on the EPDS or a score of 10–15 on the PHQ-9, four guidelines (67%) recommended discussing the specific mental health concerns and symptoms identified in the screening tool to confirm the diagnosis. For more severe scores, defined as provider concern for suicidal ideation, risk of harm, or severe mental illness, three guidelines (50%) recommended an integrated frontline or emergency mental health provider deliver immediate triage to confirm diagnosis. Only one of the nine guidelines provided recommendations for both low- and high-risk screening results.

#### Assessment by instrumentation

Two guidelines (22%) recommended a full psychosocial assessment or referral to the woman’s general practitioner if she met criteria based upon the PHQ-2; this was irrespective of severity. Nearly half of the guidelines (n = 4, 44%) specified that the EPDS should be repeated within 2–4 weeks if the respondent scored a 10 or higher to confirm diagnosis, and a high EPDS score warranted further assessment and a referral to a primary care provider or mental health professional to diagnose. The remaining three guidelines (33%) did not offer a recommendation as to which method of assessment to use.

### Summary of literature review

Although it is widely recognized that a positive screening result does not qualify as a diagnosis, little evidence was found supporting an assessment to confirm diagnosis, leading to nearly 50–70% of women remaining undiagnosed [24]. Milgrom and Gemmill [47] defined best practice as systematically following every positive screen with an offer of a diagnostic assessment. However, they cautioned that no evidence is available to support or refute this practice [47]. In addition, research is limited concerning standardized methods to diagnose PND, although several sources note that it would be feasible to incorporate assessments to confirm diagnosis into perinatal care [48, 49].

## Suicide assessment

### Guideline recommendations

Thirteen guidelines (52%) addressed suicide assessment; of them, six (46%) recommended an immediate evaluation, such as a comprehensive suicide risk assessment (SRA), in an emergency setting or by a crisis team. More than half of the guidelines (n = 7, 54%) recommended referring the respondent to a mental health specialist or psychiatrist and closely monitoring the woman’s well-being in the case suicidal risk was present. The remaining 12 guidelines (48%) did not address suicide assessment or make a recommendation for the management of suicide.

### Summary of literature review

The literature cited the importance of an emergency management protocol for ensuring women with a positive screening result for suicidal ideation are engaged in care; protocols may include: (1) medical providers verifying suicidality, (2) triggering emergency psychiatric consultation, treatment, transport (by ambulance), or admission, (3) facilitating open communication between the perinatal care team and the psychiatric team and defining the respective roles of all members, and (4) identifying medication, resources, support staff and family, and other tools needed by personnel at each stage [36, 64]. However, no evidence was found concerning the overall management of suicidality, including implementation, and its impact upon reducing suicide risk.

## Treatment

### Guideline recommendations

Almost all of the guidelines (n = 21, 84%) included treatment recommendations, with most (n = 20, 95%) providing multiple recommendations. Further, 14 guidelines (67%) offered recommendations by severity. See in Table 4.

**Table 4:** Treatment guidelines

Guideline	Treatment (intervention; provider)						
	Severity levels					Across severity levels	Alternative treatment
	Indeterminate to Mild	Mild to Moderate	Moderate to Severe	Severe	Suicidal risk		
1. Alhusen and Alvarez [6]	N/A	N/A	N/A	N/A	N/A	•Psychoeducation (N/A; unspecified) •Psychotherapy (CBT, IPT, group therapy; unspecified) •Pharmacology (SSRIs; unspecified)	N/A
2. Austin et al. [11]	•EPDS (> 10) •Psychoeducation (N/A; PCP or referral)	•EPDS (10–12) •Psychotherapy (CBT, IPT, PDT, non-directive counseling; PCP or referral)	N/A	•EPDS (12–13) •Combined psychotherapy (CBT, IPT, PDT) and pharmacology (SSRIs; PCP and referral)	N/A	N/A	N/A
3. Austin et al. [10]	N/A	•Undefined •Psychoeducation (N/A; PCP and referral) •Psychotherapy (directive counseling; PCP and referral)	•Undefined •Combined psychotherapy (unspecified) and pharmacology (SSRIs; PCP and referral)	•EPDS (> 13) •ECT (N/A; referral)	•ECT (N/A; referral)	N/A	•Electronic mental health support (apps or moderate online forums; self-administered)
4. Brock [16]	N/A	N/A	N/A	N/A	N/A	•Psychotherapy (unspecified; unspecified) •Pharmacology (unspecified; unspecified)	N/A
5. Chua et al. [22]	N/A	•Undefined •Psychoeducation (N/A; referral) •Psychotherapy (CBT, IPT; referral)	•Undefined •Pharmacology (unspecified; referral)	•Undefined •ECT (N/A; referral)	N/A	N/A	N/A
6. Committee on Obstetric Practice [23]	N/A	N/A	N/A	N/A	N/A	•Psychoeducation (N/A; unspecified) •Psychotherapy (unspecified; unspecified)	N/A
7. Curry et al. [26]	N/A	N/A	N/A	N/A	N/A	•Psychotherapy (CBT, IPT; unspecified)	N/A
8. Earls et al. [29]	•Undefined •Psychoeducation (N/A; PCP)	N/A	N/A	N/A	N/A	•Psychoeducation (N/A; referral) •Psychotherapy (unspecified; referral) •Pharmacology (unspecified; referral)	N/A
9. MacQueen et al. [46]	N/A	•Undefined •Psychotherapy (CBT, IPT; unspecified)	•Undefined •Pharmacology (unspecified; unspecified) •ECT (N/A; unspecified)	•Undefined •ECT (N/A; referral)	N/A	N/A	N/A
10. Molenaar et al. [50]	N/A	•Undefined •Psychotherapy (CBT, IPT; unspecified)	N/A	•Undefined •Pharmacology (unspecified; unspecified)	N/A	N/A	N/A
11. NICE [52]	N/A	•Undefined •Psychotherapy (CBT, IPT; referral)	•Undefined •Combined psychotherapy (CBT, IPT) and pharmacology (SSRIs, TCAs, SNRIs; referral)	•Undefined •ECT (N/A; referral)	N/A	N/A	N/A
12. Nordeng and Jettestad [53]	N/A	•Undefined •Psychotherapy (CBT, family therapy; unspecified)	N/A	N/A	N/A	•Psychotherapy (CBT, family therapy; unspecified)	N/A
13. O’Hara and Engeldinger [55]	N/A	N/A	N/A	N/A	N/A	•Psychotherapy (CBT, IPT, BA; referral) •Pharmacology (SSRIs, TCAs, SNRIs, NaSSAs; unspecified)	•Electronic mental health support (apps; self-administered)
14. Rafferty et al. [57]	N/A	•Undefined •Psychoeducation (N/A; referral) •Psychotherapy (CBT; referral)	•Undefined •Pharmacology (SSRIs; referral)	N/A	N/A	•Psychoeducation (N/A; referral)	N/A
15. RNAO [58]	N/A	•Undefined •Psychotherapy (CBT, IPT; referral)	•Undefined •Pharmacology (unspecified; PCP)	•Undefined •Combined psychotherapy (CBT, IPT) and pharmacology (unspecified; PCP or referral)	N/A	N/A	•BLT (N/A; self-administered)
16. Robson et al. [59]	N/A	N/A	N/A	•Undefined •Combined psychotherapy (CBT, IPT, PDT) and pharmacology (SSRIs; PCP and referral)	N/A	N/A	N/A
17. SIGN [63]	N/A	•Undefined •Psychotherapy (CBT, IPT, PDT; PCP or referral)	•Undefined •Pharmacology (SSRIs, TCAs; PCP and referral)	N/A	N/A	N/A	N/A
18. Siu et al. [65]	N/A	N/A	N/A	N/A	N/A	•Psychotherapy (CBT; unspecified) •Pharmacology (unspecified; unspecified)	N/A
19. Sparks et al. [68]	•PHQ-9 (5–9) •Psychoeducation (N/A; PCP or referral)	•PHQ-9 (10–14) •Psychotherapy (CBT, group therapy; referral) •Pharmacology (unspecified; PCP or referral)	•PHQ-9 (15–19) •Combined psychotherapy (CBT) and pharmacology (unspecified; PCP and referral)	•PHQ-9 (20–27) •Combined psychotherapy (CBT) and pharmacology (unspecified; PCP and referral)		N/A	N/A
20. Williams et al. [73]	N/A	•Undefined •Psychoeducation (N/A; PCP or referral) •Psychotherapy (CBT, IPT, PDT, group therapy, family therapy; PCP or referral)	•Undefined •Pharmacology (unspecified; PCP and referral) •ECT (N/A; referral)	N/A	•ECT (N/A; unspecified)	N/A	•BLT (N/A: self-administered)
21. Yonkers et al. [76]	N/A	N/A	N/A	N/A	N/A	•Psychotherapy (CBT, IPT, PDT; referral) •Pharmacology (unspecified; referral)	N/A

*BA* behavioral action; *BLT* bright light therapy; *CBT* cognitive behavioral therapy; *ECT* electroconvulsive therapy; *IPT* interpersonal psychotherapy; *NaSSAs* noradrenergic and specific serotonergic antidepressants; *PCP* primary care provider; *PDT* psychodynamic therapy; *SSRI* selective serotonin reuptake inhibitor; *SNRI* serotonin and norepinephrine reuptake inhibitors; *TCAs* tricyclic antidepressants

#### Indeterminate to mild severity

Indeterminate to mild severity was defined by a PHQ-9 score of 5–9, an EPDS score of less than 10, or undefined. Three guidelines (21%) recommended supportive care, including psychoeducation and emotional support provided in primary or obstetric care or a community health setting. Antidepressants or psychotherapy were not recommended for this severity level.

#### Mild to moderate severity

Mild to moderate depression was either undefined or defined by a PHQ-9 score of 10–14 or EPDS score of 10–12. Twelve guidelines (86%) recommended treating mild to moderate symptoms with psychotherapy, such as cognitive behavioral therapy (CBT), interpersonal psychotherapy (IPT), psychodynamic therapy (PDT), non-directive counseling in home visits, family therapy, and other counseling, in group or individual format. Treatment was recommended to be conducted through referral to a secondary mental health professional (n = 5, 42%), either a referral or the clinician (n = 3, 25%), or the clinician and an integrated mental health specialist (n = 1, 8%). Three guidelines (25%) did not specify a treatment setting. Additionally, four of the twelve guidelines (33%) recommended pairing psychotherapy with psychoeducation. Although most guidelines cited that medication is not necessary, one guideline (8%) suggested shared-decision making around using pharmacology treatment.

#### Moderate to severe

Nine guidelines (64%) provided recommendations for moderate to severe depression, which was either undefined or defined by PHQ-9 score of 15–19. Six guidelines (67%) recommended pharmacology as the first-line of treatment, and of them, selective serotonin reuptake inhibitors (SSRIs) were the most commonly cited (n = 2, 33%), followed by tricyclic antidepressants (TCAs; n = 1, 17%). Medication should be highly considered if she has expressed preference for them, has had previous positive response to medications, or has declined psychotherapy [22], National Institute for Health and Care Excellence [52]; Scottish Intercollegiate Guidelines [63]. Guidelines recommended a referral for pharmacological treatment, with two guidelines (33%) recommending the mental health specialist be in contact with the clinician around treatment. Noteworthy, one guideline (17%) recommended the primary clinician prescribe medication. Electroconvulsive therapy (ECT) was recommended by two of the six guidelines (22%) during pregnancy and/or postpartum period if the woman was unable to tolerate or take medications. ECT may be delivered by referral (n = 1, 50%) or did not specify who may deliver the treatment (n = 1, 50%).

Three guidelines (33%) recommended a combination of psychotherapy, such as cognitive behavioral therapy (CBT) or interpersonal psychotherapy (IPT), and pharmacology, such as selective serotonin reuptake inhibitors (SSRIs), tricyclic antidepressants (TCAs), and serotonin and norepinephrine reuptake inhibitors (SNRIs). Although medication alone was recommended as an alternative if psychotherapy was unavailable, psychotherapy alone was not recommended. This combined therapeutic and antidepressant treatment course is recommended to be delivered through a referral while collaborating with the clinician (n = 2, 67%) or referral alone (n = 1, 33%).

#### Severe

Severe depression was either undefined or defined as a PHQ-9 score of 20–27, EPDS score of 12–13, or EPDS score of greater than 13. Of the nine guidelines (64%) that provided recommendations for severe symptoms, four (44%) recommended a referral to electroconvulsive therapy (ECT) and four (44%) recommended a combined psychotherapy (e.g., CBT, IPT, PDT) and pharmacology (e.g., SSRIs) treatment through a referral or collaboration between provider and mental health specialist, followed by pharmacology alone (n = 1, 11%).

#### Severe with suicidal risk

Two guidelines (14%) recommended electroconvulsive therapy (ECT) during pregnancy and/or postpartum period if there was a high risk of suicide. Guidelines recommended ECT be provided by perinatal psychiatrist or in a hospital setting.

#### Across severity levels

Ten guidelines (48%) made recommendations irrespective of severity, with the majority (n = 7, 70%) making multiple recommendations. Nine of the ten (90%) recommended a referral to psychotherapy or psychosocial interventions such as cognitive behavioral therapy (CBT), interpersonal psychotherapy (IPT), psychodynamic therapy (PDT), and behavioral activation (BA), followed by six (60%) that recommended pharmacological treatment, such as selective serotonin reuptake inhibitors (SSRIs), tricyclic antidepressants (TCAs), serotonin and norepinephrine reuptake inhibitors (SNRIs), and noradrenergic and specific serotonergic antidepressants (NaSSAs), and four (40%) that recommended psychoeducation throughout the perinatal period to provide support, demystify PND, and create shared decision making around treatment options.

#### Alternative treatments

In addition to evidence-based treatments such as pharmacology and psychotherapy or psychosocial interventions**,** four guidelines (19%) recommended alternative treatments, such as Bright Light Therapy (BLT) or electronic mental health support, such as self-guided applications and moderated online forums.

### Summary of literature review

The literature on treatment types for PND was inconsistent. A systemic review of 32 studies on various psychotherapies, antidepressants, and collaborative-care treatments for pregnant and postpartum women found cognitive behavioral therapy (CBT) and related behaviorally based approaches improved depressive symptoms in postpartum women [54]. On the other hand, a meta-analysis of 27 studies on the efficacy of pharmacologic and psychological interventions for treatment found that treatments with interpersonal therapy components to have greater effect sizes than treatments with cognitive-behavioral components, and individually administered therapeutic interventions were more effective than a group format [67]. Letourneau et al.’s [43] systematic review of 36 trials of psychotherapy treatments found that although promising findings exist for IPT, CBT, and other interventions, they were unable to draw definitive conclusions regarding any one treatment that shows the most potential to influence maternal and infant outcomes. O’Conner et al. [54] also found the use of second-generation antidepressants during pregnancy may be associated with increased risk of serious maternal and infant harms to both of their physical health, while MacQueen et al. [46] found data for effectiveness of SSRI during the postpartum period. Sockol et al. [67] reported no findings on pharmacological treatments, since compared with psychological interventions there is little assessment of the efficacy of antidepressant medication. Similar to the literature on screenings, the number of included studies was relatively small, particularly for analysis of controlled effect sizes, and the quality of studies was limited [43, 46, 54, 67].

## Reassessment

### Guideline recommendations

Slightly more than a tenth of the guidelines (n = 3, 12%) provided recommendations pertaining to reassessment. Two guidelines (50%) recommended repeat screenings during pregnancy and the first postnatal year, during routine maternal and infant check-ups if clinically indicated. One guideline (25%) recommended reassessment via screening by a mental health provider.

### Summary of literature review

Limited literature was available on reassessment. One study on PND screening found a correlation between early and late EPDS scores for ‘low-risk’ women (EPDS < 10), concluding that these women may not need to be rescreened, and the limited available resources should be redirected from screening ‘low-risk’ women multiple times, towards provision of follow-up for the smaller number of women at highest risk [38]. No literature was found for other methods of reassessment.

## Remission

### Guideline recommendations

Two guidelines (8%) referenced remission, with one defining remission as the goal for treatment, which can be achieved through a PHQ-9 score less than 5. The other guideline explained that remission can be achieved through combining screenings and treatment with adequate support systems.

### Summary of literature review

A review of 32 studies found that remission was defined in various ways, including based on diagnostic criteria using the Diagnostic and Statistical Manual of Mental Disorders (DSM–5) and cutoffs advised by instruments, such as PHQ-9 score of less than 5 or EPDS score of less 10 [24]. No other literature was available on remission, including evidence that screening or reassessing for remission was the outcome for PND.

## Treatment of refractory depression

### Guideline recommendations

Twelve guidelines (48%) provided recommendations in the case that PND was not responsive to treatment, with two guidelines (17%) citing multiple recommendations. Electroconvulsive therapy was recommended by half of the guidelines (n = 6, 50%) if PND was not responsive to one or more trials of antidepressants of adequate dose and duration, followed by four guidelines (33%) which recommended pharmacologic treatment if symptoms were not responsive to psychotherapy. Two guidelines (17%) recommended combining psychotherapy and pharmacologic treatment as a second-line of treatment for those with treatment resistant depression. Finally, two guidelines (17%) gave a general recommendation to review the care plan in collaboration with the woman and support network until therapeutic goals were met.

### Summary of literature review

No literature was identified on treatment adjustment for PND that was not responsive to pharmacology or psychotherapy.

## Care management

### Guideline recommendations

Care management of PPD was discussed by more than half of the guidelines (n = 13, 52%), with one guideline (8%) making multiple recommendations. The majority (n = 11, 85%) recommended PPD be managed through a collaboration between the pediatrics or obstetric care team and mental health specialists to connect families to supportive community resources, and to refer parents for additional treatment when indicated. Only three guidelines (27%) briefly described methods to do so, such as close networks or linkages with the primary or obstetric care and mental health providers, joint case management, or Patient Centered Medical Home (PCMH). Three guidelines (23%) mentioned integrating mental or behavioral health teams into collaborative care model (CCM), however no further details were provided.

### Summary of literature review

Limited research was found for care management. Grote et al. [33] collaborative care intervention in obstetric care, MOMCare, demonstrated a trend toward reducing depression severity compared to the usual care. The intervention MOMCare also resulted in higher rates of antidepressant use and reported greater levels of satisfaction with depression care received compared with the usual care group [33]. No other evidence was found on care management, whether integrative or collaborative, specific to the perinatal period.

## Discussion

The purpose of this review was to examine best practices for the management of perinatal depression during pregnancy and the postpartum period as advised by 25 guidelines, and the evidence supporting guideline recommendations. Several findings warrant discussion.

Primarily, all of the guidelines were published within the last 12 years, although the inclusion criteria spanned the past 30 years. The recency of the publications suggests that there is increasing interest by professional associations, states, countries, and providers to identify, address, and manage PND. This is further evidenced by the sheer number of guidelines and organizations producing recommendations, as compared to previous guideline reviews such as Lewandowski et al. [44] review of adolescent depression that contained only eight guidelines. Notably, the mode publication year is 2018, raising a question of what occurred at that time to produce such a large number of publications. As anticipated, the guidelines all originated from high-income countries, as low- and middle-income countries may not have the capacity to manage perinatal depression in traditional healthcare settings due to the severe workforce shortage of professional providers often leading healthcare models to rely on lay health workers [12].

Overall, there is either little guidance available or recommendations varied on the management of PND from screening to treatment remission offered by the guidelines. Treatment was the most highly reported step in the pathway for managing PND, followed by screening. However, there were significant gaps that remained unaddressed particularly with respect to confirmation of depression diagnosis, management of suicidal risk, reassessment, and guidance for remission. Findings suggest that best practices vary greatly and often contradict one another. There are also significant issues around lack of specificity, including in areas that have the most guidance such as treatment and screening. For example, the recommended instrument, time interval, and settings for screening are inconsistent across guidelines. Although publishers focus their guidance based on their target audience and as such are unlikely to provide recommendations for screenings in settings outside of their field of practice, inconsistencies in screening settings were still found in guidelines that span the entire perinatal period from pregnancy to postpartum. Guidance around treatment often fails to specify how severity levels were defined or why they often overlapped in the score range. Our review found other inconsistency and ambiguity: some guidelines provided recommendations based on severity level or range, while others were issued irrespective of severity. This inconsistency across guidelines in the basic steps in the pathway to care suggest strongly the need for further research to solidify the evidence about the steps in the pathway, or at minimum build consensus around guidelines. Variability around who administers treatment was also found. Guidelines about recommendations on reassessment contradicted one another about the method, timing, and provider required to administer the assessment; confirmation of diagnosis similarly contained wide variability on method. The lack of guidance on confirming a diagnosis raises concern of how the provider proceeds with care without a diagnosis. Treatment for refractory depression varies based on impact and type of first-line of treatment. The lack of guidance on remission raises concerns of how management of PND is achievable without a clear path to ameliorate symptoms [19].

Across all areas, there is inadequate or contrasting evidence to support these guidelines, with a limited number of studies, most of which have small sample sizes or do not have high enough quality to meaningfully inform clinical practice. With little guidance for providers, it is unsurprising that many feel that management of PND is beyond their scope of practice, as they are unprepared with the proper skills and resources needed [19].

Throughout the guidelines, the role of the physician was often to screen, provide psychoeducation, and refer for services. However, it is unlikely that women would access services with referrals being offsite, as referrals alone have not been shown to translate to treatment engagement [60]. Although the guidelines acknowledge the need to collaborate with mental health professionals or integrate mental health services into their practice, there was minimal indication on how to do so. This is further evidenced by providers describing working in isolation from mental health professionals, despite being aware of the need to better integrate perinatal and mental health care to create a more holistic approach to care [19]. The lack of communication between these provider groups, often due to lack of access to medical records, is noted through limited feedback perinatal health care professionals receive from mental health providers about the mental health assessment and treatment plan [19].

## Limitations

This study has several limitations to be noted. There was not a systematic review of the literature on evidence for the management of perinatal depression, warranting a more comprehensive systematic search. Additionally, for the review of the evidence, only systematic reviews, and in a few cases experimental studies, were examined. The grey literature was not reviewed, indicating that there may be additional methods to manage depression that may not have been considered.

## Implications for practice

There are a wide-range of barriers for providers to address PND, including limited time and capacity, lack of available mental health specialists, dearth of materials and resources available, and inadequate mental health training [19]. These factors lead to lowered motivation and confidence to attend to women’s mental health issues [19]. Despite the acknowledged challenges, providers are motivated to learn needed skills, as they recognize the importance of addressing PND, through targeted trainings [19].

A systematic review of 1790 articles identified seven studies on strategies for professional development of health-care providers in perinatal depression [42]. Legere et al. [42] found that although the professional development interventions were diverse, the majority focused on promoting identification of perinatal depression and demonstrated modest effectiveness in improving various outcomes.

Addressing this gap requires innovative strategies to facilitate engagement, such as co-location of services. The Massachusetts Child Psychiatry Access Project (MCPAP) initiative, MCPAP for Moms, provides real-time telephone-based psychiatric consultations and care coordination for frontline providers and linkages to community-based resources [20]. Alternatively, the use of peer-led interventions may be explored, such as Screening, Education, and Empowerment (SEE) program, which is a detection and active outreach intervention for primarily poverty-impacted female caregivers who are at risk for depression led by a non-mental health paraprofessionals [2, 3]. While optimally building out the capacity for services to be offered in-house through models such as CCM, there is minimum need for consistent guidelines to inform clinical practice.

## Conclusion

In sum, our review revealed lack of consensus across guidelines on specific issues relating to management of depression during the perinatal period. This is unfortunate given the high impact of lack of care during this period for both mothers and infants. Nevertheless, there was consensus on the key steps in the pathway to care, an important finding suggesting that the critical domains of care are recognized and agreed-upon. The inconsistencies with respect to instrumentation and recommendations suggest that clinicians may use these recommendations to guide their practice, but they should remain alert to new evidence that may modify the guidelines, and they should be aware of the limitation of the evidence. Findings identify the need for substantial attention by funders of research to prioritize this area in order to develop evidence-based guidelines for managing perinatal depression.

## Data Availability

Data sharing is not applicable to this article as no datasets were generated or analyzed during the current study.

## Abbreviations

AAP: American Academy of Pediatrics
ACOG: American College of Obstetricians and Gynecologists
BA: Behavioral activation
BLT: Bright light therapy
CBT: Cognitive behavioral therapy
CCM: Collaborative care model
DSM: Diagnostic and statistical manual of mental disorders
ECT: Electroconvulsive therapy
EPDS: Edinburgh Postnatal Depression Scale
GIN: Guideline International Network
IPT: Interpersonal psychotherapy
MCPAP: Massachusetts child psychiatry access project
MDD: Major depressive disorder
NaSSA: Noradrenergic and specific serotonergic antidepressants
NCQA: National Committee for Quality Assurance
PCMH: Patient Centered Medical Home
PDT: Psychodynamic therapy
PHQ: Patient Health Questionnaire
PND: Perinatal depression
PPD: Postpartum depression
PSI: Postpartum support international
SEE: Screening, education, and empowerment program
SNRI: Serotonin and norepinephrine reuptake inhibitors
SRA: Suicide risk assessment
SSRI: Selective serotonin reuptake inhibitors
TCA: Tricyclic antidepressants
USPSTF: United States Preventive Services Task Force
